# The Switch between Protective and Nonprotective Autophagy; Implications for Autophagy Inhibition as a Therapeutic Strategy in Cancer

**DOI:** 10.3390/biology9010012

**Published:** 2020-01-04

**Authors:** David A. Gewirtz

**Affiliations:** Departments of Pharmacology and Toxicology and Medicine, Massey Cancer Center, Virginia Commonwealth University, Richmond, VA 23298, USA; David.gewirtz@vcuhealth.org; Tel.: +1-804-828-9523

**Keywords:** cytoprotective autophagy, autophagic switch, nonprotective autophagy

## Abstract

Autophagy, a process of cellular self-degradation and cell survival whereby the cell generates energy and metabolic intermediates under conditions of stress (i.e., nutrient deprivation), is also commonly induced in tumor cells in response to chemotherapy and radiation. While chemotherapy-induced autophagy and radiation-induced autophagy are generally considered to have cytoprotective functions, thereby reducing tumor cell sensitivity (and potentially conferring resistance) to various treatment modalities, autophagy can also be nonprotective; furthermore, the nature of the autophagy can be altered via the “autophagic switch” depending on such factors as the p53 status of the tumor cells. Defective or compromised autophagy has also been associated with neurodegenerative diseases, raising concerns as to the impact of autophagy inhibition on normal tissue function. Furthermore, the impact of autophagy inhibition on the immune system response to therapy as well as the influence of autophagy inhibition in combination with chemotherapy or radiation on critical tissue sites such as the bone marrow remain uncertain. These are factors requiring serious consideration within the context of current clinical efforts to exploit autophagy inhibition as a therapeutic strategy in cancer.

## 1. Pharmacologic Autophagy Inhibitors in Clinical Trials

Over the course of the last decade, one strategy that has been considered to have the potential to both enhance the response to cancer therapeutics and also to overcome drug and radiation resistance is that of autophagy inhibition. Preclinical studies of therapy-induced autophagy have generally shown autophagy to have a cytoprotective function [1,2,3,4]; cytoprotective autophagy has been demonstrated quite extensively in studies wherein autophagy inhibition promotes (apoptotic) cell death by cancer therapeutics. Consequently, it has been quite logical to infer that autophagy inhibition would result in an enhancement of therapeutic sensitivity in patients, paralleling the outcome in cell culture and tumor-bearing animal studies. However, in order for the current clinical trials combining both conventional and targeted therapies with pharmacological autophagy inhibition (i.e., using hydroxychloroquine), to have a likelihood of demonstrating efficacy, therapy-induced autophagy would have to be *exclusively* cytoprotective in function; alternatively, patients would have to be stratified based on which patients’ tumors were undergoing protective autophagy and in response to which therapeutic agents, which is currently neither practical nor feasible. Another possibility is that the pharmacologic autophagy inhibitors would have to be capable of sensitizing tumor cells to cancer therapeutics through autophagy-independent pathways.

## 2. Potential Deficiencies in Current Clinical Trials of Autophagy Inhibitors

An additional concern regarding the current clinical trials is that their outcome is entirely dependent on the chloroquine or hydroxychloroquine actually achieving levels in the patients’ tumors sufficient to inhibit autophagy, a critical indicator that we currently have no way of determining. However, even assuming that novel pharmacological autophagy inhibitors currently in development can actually suppress autophagy in the tumor cell, autophagy inhibition will not consistently achieve the desired therapeutic outcomes because of the fact that autophagy *is not uniformly cytoprotective*. Therapy-induced autophagy can also be cytotoxic [5], in which case, of course, autophagy inhibition would actually interfere with therapeutic drug effectiveness. However, this has generally not been found to be the case for most drugs currently in clinical use. Consequently, it is the *nonprotective* function of autophagy, as defined in the next paragraph [5,6,7,8,9,10], that is likely to have the most direct influence on the capacity of autophagy inhibitors to improve the therapeutic response.

## 3. The Non-Protective Form of Autophagy

It must again be acknowledged that extensive data in the preclinical literature does largely support the concept of cytoprotective autophagy as a response to cancer therapeutics in the tumor cell. Specifically, studies have shown that either pharmacological inhibitors of autophagy (such as chloroquine, bafilomycin, or 3-methyladenine) or genetic inhibition of autophagy through the knockdown or silencing of autophagy-regulatory genes, often results in an enhanced tumor response to various therapeutic modalities, both in cell culture and in tumor bearing animal studies [1,2,3,4]. However, there is also currently clear evidence for what we have termed the “nonprotective’ function of autophagy; here, neither pharmacological nor genetic autophagy inhibition produces a discernible influence on the therapeutic response [5,6,7,8,9,10]. Where this would be the case in patients, autophagy inhibition would prove to be essentially useless in the therapeutic setting.

## 4. How Does Autophagy Protect the Tumor Cell from Chemotherapy and Radiation?

It should further be emphasized that while the cytoprotective function of autophagy is intuitively understood as providing energy and metabolic intermediates necessary for cell survival under conditions of nutrient deprivation, the mechanistic basis for the protective function of autophagy in the case of radiation or chemotherapy has not been conclusively defined. Although these are clearly forms of stress that are in some ways analogous to what may be occurring in cells under nutrient deprivation, it is not immediately clear that the tumor cell requires the generation of energy or metabolic intermediates under the diverse range of therapeutic stresses induced by different forms of chemotherapy or by radiation. It is certainly feasible that a central function of autophagy in these situations is to provide protection against therapy-induced cell killing, since one primary outcome of the inhibition of protective autophagy is the promotion of apoptosis [11,12]. Consequently, autophagy could be providing an intrinsic escape from signaling pathways that would otherwise drive the demise of the cell, and could subside once the therapeutic challenge has been relieved. However, the fact that even the “classical” cytoprotective form of autophagy in the case of cancer chemotherapeutic drugs or radiation is not fully understood makes it extremely challenging to elucidate the factors that promote the protective versus the nonprotective forms of autophagy.

## 5. Autophagy in the Context of Drug and Radiation Resistance

As mentioned above, in addition to simply enhancing sensitivity to chemotherapy or radiation, autophagy inhibition has also been considered as one possible solution to overcoming drug and radiation resistance. This premise is based, in large part, on observations in the literature where tumor cells selected for drug resistance have been shown to regain sensitivity with pharmacologic or genetic autophagy inhibition [1,2,3]. It should be noted that overcoming actual resistance to radiation has generally been more difficult to demonstrate, since unlike the case with chemotherapeutic drugs, it has not proven straightforward to select for radiation resistant tumor cells in the laboratory. In any case, there is an intrinsic and fundamental problem with the premise that autophagy induction may, of itself, confer drug and radiation resistance. Hundreds of studies in the literature have demonstrated that virtually every form of cellular stress, including cancer chemotherapy and radiation, promotes autophagy. Consequently, it cannot logically follow that every experimental model wherein autophagy is induced reflects a resistance phenotype. Although it might be speculated that there exist certain unique forms or subtypes of protective autophagy that actually confer drug resistance, these is little direct experimental evidence in support of this premise.

## 6. Autophagy and Normal Tissue Function

Another factor that has perhaps been given inadequate consideration in the scientific literature is whether autophagy inhibition, even if transient, might have substantive toxicity to normal tissue function. There is considerable evidence that autophagy may serve a fundamental homeostatic function, perhaps most dramatically in the central nervous system. In fact, defects in autophagy have been implicated in neurological disease such as Parkinson’s and Alzheimer’s [13,14]. A related concern is the possibility that autophagy inhibition might exacerbate drug toxicity to e.g., the bone marrow, and/or other critical tissue sites that limit the dose and duration of cancer therapies. However, we have been unable to locate any preclinical studies that address the potential impact of autophagy inhibition on e.g., the maintenance of bone marrow function. Consequently, the generally limited patient toxicity observed throughout the current clinical trials could indicate that chloroquine and hydroxychloroquine actually fail to achieve circulating concentrations that can systemically suppress autophagy and, by extension, inhibit autophagy in the tumor cells.

## 7. Potential Influence of Autophagy Inhibition on Immune System Function

Another quite fundamental issue that should be considered within the framework of preclinical studies involving chemotherapy and radiation involves the potential influence of autophagy inhibition on the immune system. The bulk of the preclinical literature wherein autophagy inhibition has been examined within the context of cancer chemotherapeutic drugs or radiation has involved studies in immune deficient mice. This approach unfortunately ignores the potential influence of autophagy inhibition on the immune system, whether contributing to drug effectiveness or acting as a countervailing force. One of the few studies that has addressed this issue directly is the seminal study reported by Michaud et al. [15], demonstrating that autophagy inhibition was an effective therapeutic strategy in an immune deficient mouse, whereas in an immune competent animal, autophagy inhibition essentially obliterated drug action. While acknowledging that these studies were performed solely using oxaliplatin and mitoxantrone, to this author’s knowledge there is no published data that would directly contradict these findings. It should further be noted that the autophagy observed in the studies by Michaud et al. would also fit our definition of the nonprotective form of autophagy, since genetic autophagy inhibition did not sensitize the tumor cells to chemotherapy (the reader is referred specifically to figure 1B in this publication).

In general, the available literature addressing the impact of autophagy and autophagy inhibition on the immune system is quite largely inconclusive. Whereas some studies have demonstrated that autophagy facilitates an immune response and immune recognition of tumor cells, others have reported the diametrically opposite outcome. For instance, one study reported that *blocking autophagy* enhanced NK cell tumor infiltration [16]; in contrast, another indicated that *induction of autophagy* increased tumor cell sensitivity to NK-mediated lysis [17]. Similarly contradictory outcomes have been generated in studies involving T cells. For instance, autophagy was reported to suppress T cell antitumor immunity (i.e., control of tumor growth in mice was enhanced when autophagy was genetically inhibited in T cells) [18], while autophagy deficiency was shown to lead to defective T cell regulatory function in another study [19]. Admittedly, all of these reports involved entirely different experimental model systems. However, given the uncertainties associated with largely inconsistent outcomes in the literature, it would be of benefit to more systematically consider the influence of autophagy inhibition outside of direct effects on tumor cells, despite the likelihood that the immune system in humans may respond differently than in mouse models.

## 8. The Autophagic Switch

Another aspect of autophagy that is likely to influence whether autophagy inhibition could have a useful therapeutic outcome relates to what we have termed the “autophagic switch” [20,21]. We have reported on experimental situations where the nature of autophagy has been altered from one functional form to another by either therapeutic manipulations, such as prior exposure of cells undergoing irradiation to vitamin D [22,23] or a change in the genetic background from a p53 wild type state to a p53 null or mutant state [21]. In related work using two models of osteosarcoma, autophagy inhibition sensitized one cell line to gemcitabine while reducing drug sensitivity in the other cell line [24].

Evidence for the “autophagic switch” has also been generated in tumor cells *in the absence of therapy*. For instance, Vera-Ramirez et al. recently reported that “autophagy inhibition does not induce apoptosis in proliferating cells…” but “impedes the survival of dormant…cells…” [25]. This could be considered an example of a switch between the protective form of autophagy in the dormant cells and the nonprotective form in the proliferating cells. In studies of an experimental model of metastasis, Barnard et al. reported that “pharmacologic and genetic autophagy inhibition were able to impede cell proliferation in culture but did not impact the development of experimentally induced…metastases [26].” Again, these findings suggest a potential switch between the protective and nonprotective forms of autophagy.

Consequently, even postulating idealized conditions where (i) the autophagy in a patient’s tumor in response to a given therapy has been identified as being cytoprotective, (ii) pharmacologic autophagy inhibitor(s) can achieve the necessary concentration in the serum, (iii) systemic autophagy inhibition does not interfere with normal tissue function or exacerbate drug toxicity to normal tissues, and (iv) autophagy inhibition does not compromise the immune antitumor response, the nature of the autophagy might be altered (“switched”) during the course of the treatment, thereby compromising or entirely negating the desired therapeutic outcome.

## 9. Tentative Conclusions

Taken together, there appear to be multiple potential limitations to the effective clinical application of autophagy inhibition to cancer therapeutics. This does not mean that this strategy should be abandoned, but only that the outcomes of the current clinical trials must be interpreted with caution. Concern remains that something less than stellar clinical outcomes [27] may poison the well for future efforts to exploit autophagy for therapeutic benefit; in fact, the current clinical trials may prove to have been premature in the absence of a deeper understanding of the complexities of therapy-induced autophagy and the ability to (i) monitor the promotion of autophagy, (ii) to define its functional form in the patient, (iii) to confirm autophagy inhibition in the patient, as well as (iv) the potential capacity of autophagy to switch its function from the cytoprotective to nonprotective form during the course of treatment.
